# 4-(But-3-yn­yloxy)-6-(4-iodo-1*H*-pyrazol-1-yl)pyrimidine

**DOI:** 10.1107/S1600536809044031

**Published:** 2009-10-31

**Authors:** Yong-Hong Li, Ru-Liang Xie, Tao Zhang, Xiang-Dong Mei, Jun Ning

**Affiliations:** a Key Laboratory of Pesticide Chemistry and Applications, Ministry of Agriculture, Institute of Plant Protection Academy of Agricultural Sciences, Beijing 100193, People’s Republic of China

## Abstract

In the title compound, C_11_H_9_IN_4_O, the dihedral angle between the pyrazole and pyrimidine rings is 6.30 (16)°. In the crystal, weak C—H⋯O inter­actions link the mol­ecules.

## Related literature

For pharmacological background, see: Ma *et al.* (2009 ▶); Shiga *et al.* (2003 ▶). 
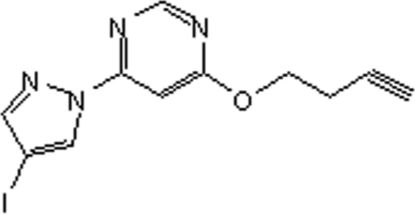


## Experimental

### 

#### Crystal data

C_11_H_9_IN_4_O
*M* *_r_* = 340.12
Monoclinic, 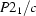

*a* = 19.511 (4) Å
*b* = 4.2670 (9) Å
*c* = 15.129 (3) Å
β = 109.18 (3)°
*V* = 1189.6 (4) Å^3^
*Z* = 4
Mo *K*α radiation
μ = 2.68 mm^−1^
*T* = 173 K
0.16 × 0.15 × 0.14 mm

#### Data collection

Rigaku MM007HF + CCD (Saturn724+) diffractometer
Absorption correction: multi-scan (*CrystalClear*; Rigaku, 2008 ▶) *T*_min_ = 0.674, *T*_max_ = 0.705
8082 measured reflections
2713 independent reflections
2577 reflections with *I* > 2σ(*I*)
*R*_int_ = 0.038

#### Refinement

*R*[*F* ^2^ > 2σ(*F* ^2^)] = 0.028
*wR*(*F* ^2^) = 0.069
*S* = 1.11
2713 reflections
154 parameters
H-atom parameters constrained
Δρ_max_ = 0.69 e Å^−3^
Δρ_min_ = −0.68 e Å^−3^

### 

Data collection: *CrystalClear* (Rigaku, 2008 ▶); cell refinement: *CrystalClear*; data reduction: *CrystalClear*; program(s) used to solve structure: *SHELXS97* (Sheldrick, 2008 ▶); program(s) used to refine structure: *SHELXL97* (Sheldrick, 2008 ▶); molecular graphics: *SHELXTL* (Sheldrick, 2008 ▶); software used to prepare material for publication: *SHELXL97*.

## Supplementary Material

Crystal structure: contains datablocks I, global. DOI: 10.1107/S1600536809044031/hb5171sup1.cif

Structure factors: contains datablocks I. DOI: 10.1107/S1600536809044031/hb5171Isup2.hkl

Additional supplementary materials:  crystallographic information; 3D view; checkCIF report

## Figures and Tables

**Table 1:** Hydrogen-bond geometry (Å, °)

*D*—H⋯*A*	*D*—H	H⋯*A*	*D*⋯*A*	*D*—H⋯*A*
C3—H3⋯O1^i^	0.95	2.40	3.249 (4)	148
C11—H11⋯N4^ii^	0.95	2.52	3.392 (4)	153

Symmetry codes: (i) 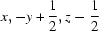; (ii) 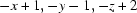.

**Fig. 1.**
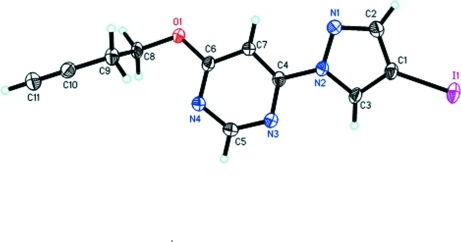
The molecular structure of (I) showing 50% displacement ellipsoids.

## supplementary crystallographic information

### Comment

Heterocyclic niteo acids and their derivatives are important starting materials in chemical synthesis. They are utilized as precursors to obtain various biologically active compounds (e.g. Ma *et al.*, 2009). Pyrazoles are an important class of compounds, which possess widespread pharmacological properties in agrochemicals (e.g. Shiga *et al.*, 2003). Pyrazolopyrimidine and related fused heterocycles are of interest as potential bioactive molecules.  Recently, we have prepared the title compound (I), which has potential herbicidal activity. The crystal structure of the title compound is shown in Fig.1. The bond lengths and angles show no unusual features.

### Experimental

The title compound (0.1 g) was dissolved in a mixed solvent of ethanol and acetone (20 ml) at room temperature: colourless blocks of (I) were obtained through slow evaporation after two weeks.

### Refinement

All the hydrogen atoms were placed at their geometrical postion with C—H = 0.93–0.98Å and *U*_iso_(H) = 1.2–1.5*U*_ep_(C).

### Crystal data

**Table:**

C_11_H_9_IN_4_O	*F*(000) = 656
*M**_r_* = 340.12	*D*_x_ = 1.899 Mg m^−^^3^
Monoclinic, *P*2_1_/*c*	Mo *K*α radiation, λ = 0.71073 Å
Hall symbol: -P 2ybc	Cell parameters from 3485 reflections
*a* = 19.511 (4) Å	θ = 2.2–27.5°
*b* = 4.2670 (9) Å	µ = 2.68 mm^−^^1^
*c* = 15.129 (3) Å	*T* = 173 K
β = 109.18 (3)°	Block, colourless
*V* = 1189.6 (4) Å^3^	0.16 × 0.15 × 0.14 mm
*Z* = 4	

### Data collection

**Table:**

Rigaku MM007HF + CCD (Saturn724+) diffractometer	2713 independent reflections
Radiation source: Rotating Anode	2577 reflections with *I* > 2σ(*I*)
Confocal	*R*_int_ = 0.038
Detector resolution: 28.5714 pixels mm^-1^	θ_max_ = 27.5°, θ_min_ = 2.2°
ω scans at fixed χ = 45°	*h* = −17→25
Absorption correction: multi-scan (*CrystalClear*; Rigaku, 2008)	*k* = −3→5
*T*_min_ = 0.674, *T*_max_ = 0.705	*l* = −19→19
8082 measured reflections	

### Refinement

**Table:**

Refinement on *F*^2^	Primary atom site location: structure-invariant direct methods
Least-squares matrix: full	Secondary atom site location: difference Fourier map
*R*[*F*^2^ > 2σ(*F*^2^)] = 0.028	Hydrogen site location: inferred from neighbouring sites
*wR*(*F*^2^) = 0.069	H-atom parameters constrained
*S* = 1.11	*w* = 1/[σ^2^(*F*_o_^2^) + (0.0244*P*)^2^ + 1.6155*P*] where *P* = (*F*_o_^2^ + 2*F*_c_^2^)/3
2713 reflections	(Δ/σ)_max_ = 0.001
154 parameters	Δρ_max_ = 0.69 e Å^−^^3^
0 restraints	Δρ_min_ = −0.68 e Å^−^^3^

### Special details

**Table:**

Geometry. All e.s.d.'s (except the e.s.d. in the dihedral angle between two l.s. planes) are estimated using the full covariance matrix. The cell e.s.d.'s are taken into account individually in the estimation of e.s.d.'s in distances, angles and torsion angles; correlations between e.s.d.'s in cell parameters are only used when they are defined by crystal symmetry. An approximate (isotropic) treatment of cell e.s.d.'s is used for estimating e.s.d.'s involving l.s. planes.
Refinement. Refinement of *F*^2^ against ALL reflections. The weighted *R*-factor *wR* and goodness of fit *S* are based on *F*^2^, conventional *R*-factors *R* are based on *F*, with *F* set to zero for negative *F*^2^. The threshold expression of *F*^2^ > σ(*F*^2^) is used only for calculating *R*-factors(gt) *etc*. and is not relevant to the choice of reflections for refinement. *R*-factors based on *F*^2^ are statistically about twice as large as those based on *F*, and *R*- factors based on ALL data will be even larger.

### Fractional atomic coordinates and isotropic or equivalent isotropic displacement parameters (Å^2^)

**Table:**

	*x*	*y*	*z*	*U*_iso_*/*U*_eq_	
I1	0.073003 (10)	0.85153 (4)	0.354299 (12)	0.02946 (8)	
O1	0.28021 (11)	−0.1265 (5)	0.89982 (14)	0.0257 (4)	
N1	0.12131 (13)	0.5308 (7)	0.63643 (17)	0.0292 (5)	
N2	0.17939 (12)	0.4249 (6)	0.61341 (15)	0.0217 (5)	
N3	0.29051 (13)	0.1759 (6)	0.65001 (17)	0.0281 (5)	
N4	0.34178 (13)	−0.1049 (6)	0.79327 (17)	0.0258 (5)	
C1	0.11287 (15)	0.6716 (6)	0.48870 (19)	0.0236 (6)	
C2	0.08146 (17)	0.6802 (7)	0.5602 (2)	0.0304 (7)	
H2	0.0368	0.7814	0.5544	0.037*	
C3	0.17555 (14)	0.5053 (7)	0.52485 (18)	0.0232 (5)	
H3	0.2097	0.4556	0.4944	0.028*	
C4	0.23353 (14)	0.2441 (7)	0.67752 (18)	0.0202 (5)	
C5	0.34119 (16)	0.0049 (8)	0.7106 (2)	0.0323 (7)	
H5	0.3823	−0.0458	0.6929	0.039*	
C6	0.28450 (14)	−0.0285 (7)	0.81731 (18)	0.0218 (5)	
C7	0.22707 (15)	0.1515 (6)	0.7616 (2)	0.0213 (5)	
H7	0.1866	0.2062	0.7801	0.026*	
C8	0.33899 (15)	−0.3164 (7)	0.9591 (2)	0.0254 (6)	
H8A	0.3574	−0.4565	0.9198	0.030*	
H8B	0.3209	−0.4486	1.0004	0.030*	
C9	0.40039 (15)	−0.1086 (7)	1.0183 (2)	0.0266 (6)	
H9A	0.4196	0.0174	0.9768	0.032*	
H9B	0.3813	0.0378	1.0554	0.032*	
C10	0.45916 (15)	−0.2943 (7)	1.0816 (2)	0.0255 (6)	
C11	0.50610 (16)	−0.4493 (8)	1.1313 (2)	0.0322 (6)	
H11	0.5439	−0.5741	1.1714	0.039*	

### Atomic displacement parameters (Å^2^)

**Table:**

	*U*^11^	*U*^22^	*U*^33^	*U*^12^	*U*^13^	*U*^23^
I1	0.03776 (13)	0.02646 (12)	0.01837 (11)	0.00202 (7)	0.00135 (8)	0.00445 (7)
O1	0.0241 (10)	0.0348 (11)	0.0192 (9)	0.0074 (8)	0.0083 (8)	0.0097 (8)
N1	0.0288 (12)	0.0389 (14)	0.0208 (12)	0.0103 (11)	0.0094 (9)	0.0032 (11)
N2	0.0217 (11)	0.0264 (11)	0.0155 (10)	0.0027 (9)	0.0039 (8)	0.0010 (9)
N3	0.0245 (12)	0.0402 (15)	0.0200 (12)	0.0071 (10)	0.0079 (10)	0.0063 (10)
N4	0.0227 (11)	0.0348 (13)	0.0193 (11)	0.0067 (9)	0.0061 (9)	0.0024 (10)
C1	0.0281 (14)	0.0230 (14)	0.0167 (13)	0.0016 (10)	0.0035 (11)	0.0009 (10)
C2	0.0287 (15)	0.0376 (17)	0.0235 (15)	0.0106 (12)	0.0063 (12)	0.0030 (12)
C3	0.0251 (13)	0.0276 (14)	0.0149 (12)	0.0001 (11)	0.0039 (10)	0.0035 (10)
C4	0.0204 (12)	0.0207 (12)	0.0167 (12)	−0.0006 (10)	0.0025 (9)	−0.0013 (10)
C5	0.0265 (14)	0.048 (2)	0.0252 (14)	0.0129 (13)	0.0124 (11)	0.0054 (13)
C6	0.0225 (12)	0.0248 (13)	0.0160 (12)	−0.0009 (10)	0.0037 (10)	0.0009 (10)
C7	0.0199 (12)	0.0253 (14)	0.0186 (13)	0.0031 (9)	0.0064 (10)	0.0015 (10)
C8	0.0262 (14)	0.0263 (14)	0.0208 (13)	0.0046 (11)	0.0041 (11)	0.0091 (11)
C9	0.0270 (14)	0.0268 (14)	0.0232 (14)	0.0032 (11)	0.0042 (11)	0.0041 (11)
C10	0.0251 (14)	0.0294 (14)	0.0221 (13)	−0.0019 (11)	0.0076 (11)	0.0010 (11)
C11	0.0270 (15)	0.0389 (17)	0.0289 (15)	0.0044 (13)	0.0066 (12)	0.0056 (14)

### Geometric parameters (Å, °)

**Table:**

I1—C1	2.071 (3)	C3—H3	0.9500
O1—C6	1.345 (3)	C4—C7	1.376 (4)
O1—C8	1.449 (3)	C5—H5	0.9500
N1—C2	1.324 (4)	C6—C7	1.391 (4)
N1—N2	1.367 (3)	C7—H7	0.9500
N2—C3	1.361 (3)	C8—C9	1.523 (4)
N2—C4	1.406 (3)	C8—H8A	0.9900
N3—C5	1.325 (4)	C8—H8B	0.9900
N3—C4	1.341 (3)	C9—C10	1.462 (4)
N4—C6	1.325 (3)	C9—H9A	0.9900
N4—C5	1.332 (4)	C9—H9B	0.9900
C1—C3	1.363 (4)	C10—C11	1.178 (4)
C1—C2	1.408 (4)	C11—H11	0.9500
C2—H2	0.9500		
			
C6—O1—C8	118.1 (2)	N4—C5—H5	115.9
C2—N1—N2	103.7 (2)	N4—C6—O1	119.6 (2)
C3—N2—N1	112.7 (2)	N4—C6—C7	123.6 (2)
C3—N2—C4	127.1 (2)	O1—C6—C7	116.8 (2)
N1—N2—C4	120.2 (2)	C4—C7—C6	114.7 (2)
C5—N3—C4	114.3 (2)	C4—C7—H7	122.6
C6—N4—C5	115.0 (2)	C6—C7—H7	122.6
C3—C1—C2	105.4 (2)	O1—C8—C9	110.4 (2)
C3—C1—I1	125.8 (2)	O1—C8—H8A	109.6
C2—C1—I1	128.8 (2)	C9—C8—H8A	109.6
N1—C2—C1	112.2 (3)	O1—C8—H8B	109.6
N1—C2—H2	123.9	C9—C8—H8B	109.6
C1—C2—H2	123.9	H8A—C8—H8B	108.1
N2—C3—C1	106.2 (2)	C10—C9—C8	111.5 (2)
N2—C3—H3	126.9	C10—C9—H9A	109.3
C1—C3—H3	126.9	C8—C9—H9A	109.3
N3—C4—C7	124.2 (2)	C10—C9—H9B	109.3
N3—C4—N2	114.6 (2)	C8—C9—H9B	109.3
C7—C4—N2	121.2 (2)	H9A—C9—H9B	108.0
N3—C5—N4	128.2 (3)	C11—C10—C9	178.6 (4)
N3—C5—H5	115.9	C10—C11—H11	180.0
			
C2—N1—N2—C3	−0.3 (3)	N1—N2—C4—C7	4.6 (4)
C2—N1—N2—C4	−178.2 (3)	C4—N3—C5—N4	0.3 (5)
N2—N1—C2—C1	0.1 (4)	C6—N4—C5—N3	−0.8 (5)
C3—C1—C2—N1	0.1 (4)	C5—N4—C6—O1	−179.6 (3)
I1—C1—C2—N1	177.7 (2)	C5—N4—C6—C7	0.3 (4)
N1—N2—C3—C1	0.4 (3)	C8—O1—C6—N4	−0.6 (4)
C4—N2—C3—C1	178.1 (3)	C8—O1—C6—C7	179.6 (2)
C2—C1—C3—N2	−0.3 (3)	N3—C4—C7—C6	−1.3 (4)
I1—C1—C3—N2	−177.96 (19)	N2—C4—C7—C6	179.4 (2)
C5—N3—C4—C7	0.8 (4)	N4—C6—C7—C4	0.7 (4)
C5—N3—C4—N2	−179.8 (3)	O1—C6—C7—C4	−179.5 (2)
C3—N2—C4—N3	7.6 (4)	C6—O1—C8—C9	84.8 (3)
N1—N2—C4—N3	−174.8 (3)	O1—C8—C9—C10	177.7 (2)
C3—N2—C4—C7	−173.0 (3)		

### Hydrogen-bond geometry (Å, °)

**Table:**

*D*—H···*A*	*D*—H	H···*A*	*D*···*A*	*D*—H···*A*
C3—H3···O1^i^	0.95	2.40	3.249 (4)	148
C11—H11···N4^ii^	0.95	2.52	3.392 (4)	153

Symmetry codes: (i) *x*, −*y*+1/2, *z*−1/2; (ii) −*x*+1, −*y*−1, −*z*+2.
